# Parental understanding of crucial medical jargon used in prenatal prematurity counseling

**DOI:** 10.1186/s12911-020-01188-w

**Published:** 2020-07-22

**Authors:** Nicole M. Rau, Mir A. Basir, Kathryn E. Flynn

**Affiliations:** 1 Department of Pediatrics, Medical College of Wisconsin, 999 N 92nd St, Suite C410, PO Box 1997, Wauwatosa, WI 53226 USA; 2 Department of Medicine, Medical College of Wisconsin, 9200 W. Wisconsin Ave. Suite C5500, Milwaukee, WI 53226 USA

**Keywords:** Jargon, Counseling, Prematurity, Literacy, Interviews, Shared decision making

## Abstract

**Background:**

Parent-clinician shared decision making is the recommended model for the care of premature infants; thus, clinicians provide prenatal prematurity counseling to parents in the event of a mother’s hospitalization for premature birth. However, parental understanding of medical jargon commonly used during prematurity counseling is unknown.

**Methods:**

Within an overall research agenda to develop and test an educational aid for prenatal prematurity education, we designed the Parental Knowledge of Premature Birth questionnaire. To evaluate parental comprehension of the medical jargon contained within the questionnaire, we conducted cognitive interviews, a formal method for evaluating comprehension and response to questionnaire items. Parents were recruited from a Level IV Neonatal Intensive Care Unit; purposeful recruitment ensured diversity with respect to gender, race, literacy level, and child’s gestational age. Data collection and analysis followed standard qualitative methods for cognitive interviewing. We report on the insights gained from these cognitive interviews regarding parental understanding of crucial medical jargon commonly used during prenatal prematurity counseling.

**Results:**

Participants included 10 women and 6 men who ranged in age from 23 to 38 years and represented Black/African-American (38%), Asian (6%), and white (56%) backgrounds. Five participants (31%) had less than a high school education or reading level below 9th grade (Wide Range Achievement Test version 4 reading subtest). In the first round of interviews, parents of all education and literacy levels had difficulty with medical jargon commonly used in prematurity counseling. Terms that parents found difficult to understand included “gestational age”, “mild or no developmental problems”, and “neonatologist”. Modified terms tested in a second round of interviews showed improved comprehension.

**Conclusion:**

Cognitive interviews provided empirical testing of parental understanding of crucial medical jargon and highlighted that language commonly used during prenatal prematurity counseling is not understood by many parents. For parents to participate in shared decision making, plain language should be used to maximize their understanding of medical information.

## Background

Parent-clinician shared decision making is the recommended model for the care of premature infants. Shared decision making is a process in which clinicians and patients work together to make decisions and select tests, treatments and care plans based on clinical evidence that balances risks and expected outcomes with patient preferences and values [1]. Examples include the decision to provide neonatal palliative care or resuscitation in cases of extremely premature birth. Clinicians and parent advocacy groups have identified information that parents experiencing premature birth need to know prenatally to make informed decisions [2, 3]. To this end, clinicians provide prenatal prematurity counseling to parents during the mother’s hospitalization for premature birth [4].

Studies evaluating this practice have identified deficiencies in how effectively information is provided to these parents. These studies point to the poor concordance between parent and provider perceptions of the prenatal consult, including the content of the consult and if a treatment plan has been made [5, 6], as well as to significant gaps in parent knowledge related to prematurity following counseling [7]. Additionally, a previously conducted national survey found that only 1% of U.S. hospitals surveyed provided specialized communication training to prematurity counselors [4]. Further complicating this interaction, 20% of US adults read at or below a fifth-grade level and more than 90 million adults have limited health literacy [8, 9]. Deficiencies in provider communication skills and low health literacy may further hamper parent understanding of prematurity information.

An expert panel convened in 2014 by the National Institutes of Child Health and Human Development (NICHD) identified the need to develop educational-aids to improve the prematurity counseling process to better prepare families for making these decisions [2]. We developed the Parent Knowledge of Premature Birth questionnaire (reported separately) to assess the efficacy of such educational-aids. As part of development of the Parent Knowledge of Premature Birth questionnaire, we conducted cognitive interviews with parents to ensure that parents understood and interpreted the questionnaire items as intended. Comprehension of the language used during a prenatal consult is clearly essential for effective prematurity counseling and parental decision making. However, to our knowledge, no studies to date have evaluated parent comprehension of medical jargon commonly used in prenatal counseling and in previously developed counseling tools. The aim of this study was to determine if parents correctly understood the medical jargon and phrasing of items contained within the Parent Knowledge of Premature Birth questionnaire. A primary modern method for evaluating whether questionnaire items are fit for use is cognitive interviewing [10]. In this manuscript, we report on the results of cognitive interviews regarding parental misunderstanding of crucial medical jargon commonly used during prematurity counseling.

## Methods

### Questionnaire item development

Separate versions of the Parent Knowledge of Premature Birth questionnaire were created for 22–24 weeks and 25–33 weeks gestational ages (GA) because different information is recommended for parents in these GA categories. The original questionnaire items were developed from published recommendations (Table 1). Items aimed to assess knowledge of GA-specific survival, short-term outcomes, long-term outcomes, treatment options, and length of stay in the Neonatal Intensive Care Unit (NICU). The 22–24 week questionnaire also addressed the concept of neonatal resuscitation. Each item was reviewed and revised by designated prematurity counselors at our institution and the study team, representing expertise in neonatology and questionnaire development. The revised items comprised the Parent Knowledge of Premature Birth questionnaire tested in our cognitive interviews. The 22–24 week version contained 43 items initially and 42 items after final revision, and the 25+ week version contained 47 items initially and 38 items after final revision.

**Table 1:** Recommendations about prenatal counseling in policy statements

**AAP Recommendation** [3]	
Discussion should be appropriate to family’s level of understanding	
Counseling should be sensitive to family’s religious, social, cultural, and ethnic diversity	
Provide the most accurate prognostic morbidity and mortality data available (local or national data)	
Discuss that despite intensive care, many extremely premature infants die in the first few days	
Parents have the option to withdraw treatment later even if resuscitation is successful	
Discuss all options for care including comfort care if appropriate	
Provide time for parents to ask questions	
Ideally OB and Neonatology will discuss resuscitation together so that consistent approach is presented to parents	
**ACOG Recommendations** [11]	
Counseling regarding short-term and long-term outcomes should take into consideration anticipated gestational age at delivery as well as other variables	
Counseling should be provided by a multidisciplinary team	
A pre-delivery plan should be made with parents but may be modified based on evolution of the clinical situation	
**NICHD Recommendations** [2]	
Counseling should be bi-directional, collaborative, and ongoing process	
Discussion of the alternative to and rationale for or against active maternal and neonatal intervention are appropriate	
Institutional, regional, or national data regarding outcomes should be provided as available	
Consider the use of decision aids or other materials	
Provide information regarding the possibility of survival and disabilities separately	
Offer information regarding anticipated NICU care and NICU complications	
Information given to families should include what some children cannot do because of disabilities and what may can do	
Discuss options for comfort care and circumstances that might result in reconsideration of life-sustaining interventions	

### Study sample

We recruited participants in person from the Level IV NICU of a Midwestern Academic Children’s Hospital. Eligible participants were 18 years or older, English-literate parents of neonates with GA between 22 weeks 0 days and 33 weeks 6 days who were currently admitted to the NICU. Purposeful recruitment used quota sampling so that at least 25% of the participants were non-white and at least 25% had low literacy, defined by less than a high school education or reading level less than ninth-grade, assessed by the Wide Range Achievement Test version 4 (WRAT-4) reading subtest administered during the cognitive interview. We included men and women. Thus, all items were reviewed by a diverse group of parents based on gender, race, and literacy level to satisfy the study objectives. Parents of 22–24 weeks premature infants tested the 22–24 week questionnaire, and parents of 25–33 weeks infants tested the 25–33 week questionnaire. Participants were compensated $50 for their time.

### Cognitive interviews

Cognitive interviewing is a qualitative method that formally evaluates questionnaire items [12]. It provides evidence for the content validity of questionnaire items through identification of hidden problems with item phrasing and word choice. Cognitive interviewing is predicated on Tourangeau’s four-stages of the cognitive response process used when answering questions: comprehension of the question, retrieval from memory of relevant information, judgment/estimation processes, and response processes [13]. We used Willis’s Question Appraisal System to identify potential sources of error within the draft items [12]. Based on this appraisal, we developed an interview guide with item-specific probes to be used during the cognitive interviews. We report on our findings guided by the Cognitive Interviewing Reporting Framework [14].

Each cognitive interview was conducted in person, in a private meeting room in the NICU. The interviewer (W.S.) had previous experience conducting cognitive interviews and was trained by the study team [15]. In addition to the interviewer, a member of the study team (N.R.) took notes. There was no existing relationship between the participants and the interviewer; the interviewer described the goals of the study before beginning the interview. All interviews were audio recorded for later review, if needed, but recordings were not transcribed verbatim. Participants first completed the questionnaire in its entirety. Each item was then evaluated using verbal probing to assess comprehension of the item (e.g., What is the question asking you? Can you rephrase it in your own words? Can you define the following term?) and response construction (e.g., How did you decide on your answer?). The interviewer used additional free form prompts related to other response processes as needed (i.e., retrieval from memory of relevant information, judgment/estimation processes). As the items did not include a recall period, we did not ask about recall. Each interview lasted about one hour.

A key premise of cognitive interviewing methodology is that questionnaire items are improved through in-depth questioning of a relatively small number of individuals, who can effectively stand in for the target survey respondents [14]. Our cognitive interviews were conducted in two rounds, with 8 participants per round. After each interview, interview notes were transferred from paper forms to an Excel spreadsheet which tracked participant comments for each item, noting the participant’s gender, literacy level, and baby’s GA. Analysis followed Willis’ qualitative reduction approach [12], wherein comments were evaluated by each member of the study team, and problems (especially with comprehension of questionnaire items) were noted, summarized across the items, and then items were revised as needed. Revised items were then retested in the second round of cognitive interviews with new participants, following the same process as round one. Final items were revised to use consistent language across the questionnaire. Participants did not provide feedback on the findings.

## Results

Twenty-one eligible parents were invited to participate. Of these, 2 declined, and 3 consented but were unable to be interviewed due to scheduling conflicts. A total of 16 cognitive interviews were conducted between March and June 2017. Table 2 shows the characteristics of the participants. The 10 women and 6 men ranged in age from 23 to 38 years old. Six participants were Black/African American (38%), 1 was Asian (6%), and 9 were white (56%). Five of the participants had low literacy (31%). Many of the tested items were uniformly understood by parents without modification. Herein we describe findings relevant to items not uniformly understood by parents. The full Parent Knowledge of Premature Birth questionnaire will be reported separately.

**Table 2:** Demographic characteristics

**Characteristic**	**All participants (*n*** **= 16)**	**Men (*n*** **= 6)**	**Women (*n*** **= 10)**
Age in years, mean (range)	29 (23–38)	31 (23–35)	28 (23–38)
WRAT-4 score,^a^ mean (range)	59 (47–69)	60 (47–69)	59 (48–67)
Low literacy,^b^ n (%)	5 (31)	2 (33)	3 (30)
Education, n (%)			
Less than high school	1 (6)	0	1 (10)
High school diploma	1 (6)	0	1 (10)
Some college	6 (38)	2 (33)	4 (40)
Bachelor’s degree	6 (38)	3 (50)	3 (30)
Advanced degree	2 (13)	1 (17)	1 (10)
Race, n (%)			
Black/African American	6 (38)	2 (33)	4 (40)
Asian	1 (6)	1 (17)	0
White	9 (56)	3 (50)	6 (60)
Gestational age at delivery, Mean (range)	27 (23–33)	27 (23–33)	26 (23–33)

^a^ WRAT-4 reading subtest score ranges from 0 to 70

^b^ Low Literacy defined as less than high school education or WRAT-4 reading subtest score < 54

### Medical jargon and high-literacy vocabulary

Through the cognitive interview process, it was discovered that several terms not considered medical jargon by the study team were difficult for parents to understand. One such term was “neonatologist”. Several participants (2/8) were unfamiliar with the term, with one asking, “what does neonatologist mean?” The second round of cognitive testing revealed that the term “baby specialist” improved parental understanding and was overall preferred by parents in round 2. Table 3 demonstrates the original items as well as how they were revised throughout the cognitive interviewing process.

**Table 3:** Example of item revision from cognitive interviewing process

**Original item presented to prematurity experts**	**Item tested in Round 1 cognitive interviews**	**Item tested in Round 2 cognitive interviews**	**Final item**
Neonatal medical team will need to be present at birth	Neonatal medical team will need to be present at birth	Baby specialists will need to be present at birth	The baby specialists will need to be present at my delivery
Parents can switch to comfort care treatment if complications happen, even if resuscitation is chosen at the beginning.	I cannot stop intensive care treatment for my premature baby if complications happen	Parents have the option to stop intensive care treatment for their premature baby if serious complications happen	Parents have the option to stop intensive care treatment for their premature baby if serious complications happen
Before 25 weeks, parents can decide if they want full resuscitation or comfort care	What is the highest gestational age when parents can choose not to use intensive care treatment at birth	What is the highest gestational age at which parents can choose comfort care at birth?	What is the highest week of pregnancy at which parents can choose comfort care at birth
By how many days can the due date change, when the due date is based on a first trimester ultrasound	When the due date is based on an ultrasound performed in the first 13 weeks (first trimester) of pregnancy, by how many days can gestational age vary?	How accurate is your baby’s gestational age if it is based on a first trimester ultrasound? Within ____ days	How accurate is your due date?
		How accurate is your due date if it is based on a first trimester ultrasound?	
		How accurate is your due date?	
May not have any long lasting problems because of prematurity	May not have any long lasting problems because of prematurity	May be healthy later in life	May be healthy as a teenager

Additional items throughout the questionnaire used the terms “gestation” and “gestational age”. One representative item asked, “What is the lowest gestational age at which a baby can survive after birth?” Three participants, two with low literacy, could provide no definition of this term. An additional 4/16 participants provided incorrect definitions like “how long after coming out of the womb” or “date of arrival”. The remaining 9/16 participants provided correct definitions including “how many weeks inside of me” and “how long it had been since she was conceived”. However, despite many participants understanding this term, the concept of weeks of gestation was a repeated source of confusion. After revision, participants preferred the term “weeks of pregnancy”, finding this easier to understand.

While making delivery room resuscitation plans, it is recommended that parents understand that the assigned GA is an estimate [2]. Excluding in-vitro fertilization (IVF), first trimester ultrasounds provide the best obstetric estimate; however, even this may vary up to five days [16]. A relevant item was “when due date is based on ultrasound done in the first 13 weeks (first trimester) of pregnancy, by how many days can gestational age vary?” Many parents (7/16) had difficulty answering this item. One parent commented that the question was “confusing, I had to re-read it a few times”. Parents expressed difficulty with the phrase “can gestational age vary?” We tested several versions of this question in the second round of interviews. Participants preferred the phrasing “How accurate is your due date?”

Prenatal counseling also includes discussion of morbidities such as intraventricular hemorrhage, bronchopulmonary dysplasia, cerebral palsy, and retinopathy of prematurity. These medical terms were identified as a source of confusion by 5/16 participants when asked if, overall, any of the questions were particularly difficult to answer. Participants whose infants had these complications were most likely to provide accurate definitions, and one father noted that he would not have understood the terms pre-delivery.

Relatedly, one item-specific probe inquired “what did you think of when you read ‘mild or no developmental problems’?” Three participants (1 with low literacy) either could provide no explanation of the term or provided an inaccurate definition. Five participants were able to provide some definition such as “some problems or none at all” or “completely fine without issues.” The remaining participants (8/16) were able to correctly define this term and provide appropriate examples such as “cognitive/motor skills that you can help” or “not on track for normal age range, little slow development but will get there.” As with the specific names of morbidities (interventricular hemorrhage, retinopathy of prematurity, etc) we choose not to remove the jargon but rather incorporated more description of the condition into the item and our educational aid.

## Discussion

The consensus statements by medical societies and the 2014 NICHD expert panel have pointed to deficiencies in the prematurity counseling process and called for improvements to better prepare families [2, 3, 5, 6, 11, 17]. One deficiency in this system may be the use of high level vocabulary, also known as medical jargon. In the United States, 20% of adults read at or below a fifth-grade level and more than 90 million adults have limited health literacy [8, 9]. However, to our knowledge, this is the first study to evaluate parental understanding of medical terms that are often used by clinicians during prematurity counseling. Cognitive interviewing methodology is an important tool to evaluate comprehension in questionnaire development [10], and through the use of cognitive interviews with parents who had experienced prematurity counseling, our findings reveal that many parents may not understand medical terms that are often used during counseling. Additionally, we have outlined our process for applying cognitive interviewing to confirm patient understanding and we recommend incorporation of this technique into development of future educational tools and patient handouts.

Advanced communication skills are essential for effective counseling [18]. In a national survey of prematurity counseling practices we previously found that only 5 out of 352 (1%) U.S. hospitals provided specialized communication training to prematurity counselors [4]. Evidence from other medical contexts evaluating patient-provider communication shows that use of plain language and simplified information improves patient comprehension, compliance with instructions, and shared decision making [8, 9, 18–20]. Clinician’s use of plain language also helps build rapport with patients by increasing the perception of professionalism and empathy [21]. However, clinicians often overestimate the clarity of the information they provide and may not recognize their use of medical jargon [22]. Recently there has been a focus on improved patient communication within medical education. Introduction of plain language education and prematurity improvisation workshops have improved clinician-patient communication and self-perceived counseling skills [9, 20, 23, 24]. Our study reveals commonly used medical jargon that counselors may not realize is confusing for parents and provides alternate terms that are simpler for parents to understand. Identification of medical jargon used during counseling and alternate plain language words will add to prematurity counselor training and improve effectiveness of this parent-clinician communication.

Limited empirical work has been conducted in the area of parent decision making. In a retrospective qualitative study, mothers (*n* = 26) said they wanted to participate in decisions regarding delivery room resuscitation but were guided more by religion, spirituality, hope, and their emotions than by physician prognoses for their premature infants [25]. Another study looking at prematurity counseling also pointed to the critical role of parents’ emotions in decisions and difficulties conveying complex medical information to parents [26]. In other medical areas, previous work has demonstrated that framing and content (medicalized v. de-medicalized) can influence treatment decisions [27]. Finally, a study by Geurtzen et al. surveyed parents and medical professionals to determine recommendations for the style, content, decision making, and organization of prenatal counseling. Recommendations included avoiding jargon, adapting language to the level of the parents, and checking for understanding [28]. Taken together, these studies highlight the large impact of word choice on parent understanding and decision making. In order to support shared decision making, providers must move to plain language discussions and check for parent understanding of the information provided.

The end users of this questionnaire and recipients of prematurity counseling will be parents who are admitted for premature labor but have not yet delivered. A limitation of this study is that we conducted our cognitive interviews with parents of infants in the NICU. These parents already have experience with a premature baby and thus may have more knowledge of prematurity than our target population. We felt that conducting cognitive interviews with mothers admitted for premature labor (but not yet delivered) would impose too high of a burden on this population and pregnant women who were not in labor would not have received prenatal counseling. The closest representation of our target population was parents who had undergone prenatal counseling and now had infants in the NICU. When this questionnaire is used with our target population, we may find additional terms that are misunderstood and need revision. An additional limitation of this study is the small sample size, which may limit its generalizability.

## Conclusions

Cognitive interviews conducted with a diverse, purposive sample of NICU parents showed that a large proportion of parents struggle with medical jargon that is commonly used during prematurity counseling. In order for parents to meaningfully participate in shared decision making regarding their infant, this study reinforces the use of plain language to maximize parental understanding of complex new medical information that is presented during prematurity counseling.

## Data Availability

The datasets used and/or analyzed during the current study are available from the corresponding author on reasonable request.

## Abbreviations

NICHD: National Institutes of Child Health and Human Development
GA: Gestational age
NICU: Neonatal Intensive Care Unit
WRAT-4: Wide Range Achievement Test version 4
IVF: In Vitro Fertilization
AAP: American Academy of Pediatrics
ACOG: American College of Obstetricians and Gynecologists
